# Minimum intervention oral care - incentivising preventive management of high-needs/high caries-risk patients using phased courses of treatment

**DOI:** 10.1038/s41415-024-7132-2

**Published:** 2024-03-08

**Authors:** ﻿Avijit Banerjee, Zain Hameed, M. Ali Chohan, Kish Patel, Jin J. Vaghela, Fahad Sheikh, Nick Barker, Pritesh Shah, Divyash Patel

**Affiliations:** 4141534836001 Professor of Cariology & Operative Dentistry and Honorary Consultant, Restorative Dentistry, Faculty of Dentistry, Oral & Craniofacial Sciences, King´s College London, UK; Honorary Consultant Advisor, Office of the Chief Dental Officer, England, UK; 4141534836002 Dental Core Trainee 2, Community and Special Care Dentistry, Barts Health NHS Trust, UK; 4141534836003 Dental Surgeon, London, UK; Clinical Advisor to NHS England, England, UK; Discipline Specific Member for the Performance Advisory Group, NHSE, UK; Clinical Advisor, General Dental Council, UK; Dental Foundation Training Educational Supervisor, Health Education, East of England, England, UK; Honorary FDS Lecturer, Royal College of Surgeons England, UK; Visiting Associate Professor, Restorative and Aesthetic Dentistry, College of Medicine and Dentistry, UK; 4141534836004 Visiting Lecturer, Eastman Dental Institute, UK; Reference Group For Professional Framework, College of General Dentistry, UK; Editorial Board, Private Dentistry, UK; CEO and Founder, Smile Clinic Group and Smile Dental Academy, London, UK; 4141534836005 Dental Surgeon, London, UK; Dental Foundation Training Educational Supervisor, Health Education, East of England, England, UK; Visiting Associate Professor, Restorative and Aesthetic Dentistry, College of Medicine and Dentistry, UK; CEO and Founder, Smile Clinic Group and Smile Dental Academy, London, UK; Fellow, College of General Dentistry, UK; Visiting Lecturer, Eastman Dental Institute and Royal College of Surgeons England, UK; 4141534836006 Dental Surgeon, London, UK; Dental Foundation Training Educational Supervisor, Health Education, East of England, England, UK; 4141534836007 Deputy Chief Dental Officer, England, UK; 4141534836008 Lead Dental Advisor, NHS England, London Region, UK; 4141534836009 Clinical Policy Lead, Office of the Chief Dental Officer England, UK

## Abstract

This paper demonstrates how person-focused, prevention-based, risk/needs-related, team-delivered, minimum intervention oral care (MIOC) principles and approaches can be integrated into the dental profession for the delivery of environmentally sustainable, optimal care to high-needs and high caries-risk/susceptibility patients. It highlights the potential for NHS remuneration for prevention-based, phased, personalised care pathways/plans (PCPs) within a reformed NHS dental contract system. It emphasises the importance of comprehensive and longitudinal patient risk/susceptibility assessments, prevention and stabilisation of the oral environment before considering more complex, definitive restorative work. This paper forms the first of several components of a suite of educational/information materials needed to instil confidence and implementation protocols within primary care clinical oral health care teams delivering MIOC through phased PCPs, especially when managing patients with high needs and/or disease susceptibility.

## Key points

- The development of a team-delivered, prevention-based, person-focused, susceptibility/needs-related phased care approach to modern caries management using the minimum intervention oral care delivery framework in primary care is proposed.
- Using personalised care plans within phased courses of treatment, aligned to periodontal management guidelines, caries prevention in primary care can be incentivised.
- The potential for NHS remuneration for prevention-based, phased, personalised care pathways/plans (PCPs) within a reformed NHS dental contract system is outlined.

## Context

Minimum intervention oral care (MIOC) delivery focuses on person-focused, risk/susceptibility-related oral and dental disease prevention and management, reliant upon longer-term preventive behaviours and actions of the patient/caregiver, guided and/or delivered by members of the oral health care team.

**Fig. 1:**
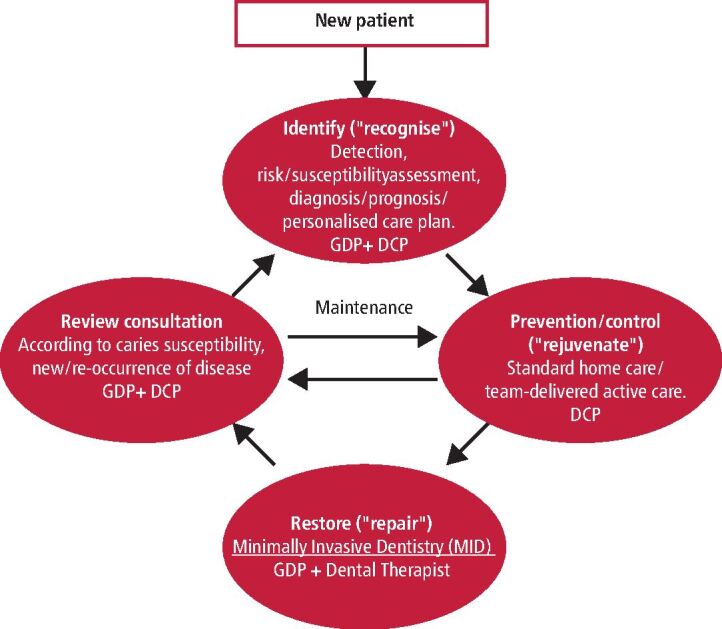
The MIOC approach showing the four interlinking clinical domains of care: disease identification/patient assessment, disease control/lesion prevention, minimally invasive operative interventions and review (recall/active surveillance). The arrows indicate the direction of patient flow through this cycle and within each domain an indication is given of the members of the oral healthcare team who might be included (GDP = general dental practitioner; DCP = dental care professional, including oral health educator-trained nurses, dental hygienists, dental therapists, practice managers, reception staff). Reproduced from A. Banerjee, ‘MI'opia or 20/20 vision?', *British Dental Journal*, Vol 214, Springer Nature, 2013,^2^ with permission from SNCSC. The licensed material is not part of the governing OA license but has been reproduced with permission

It is appreciated that there is a perception that remuneration mechanisms within NHS dentistry may limit MIOC implementation among primary care dental practice/oral health care teams, particularly when treating unmanaged generalised disease, especially in those patients presenting with high and complex oral and medical health needs. Recent NHS dental system reform and expansion of Band 2 remuneration^1^ provides an opportunity to integrate effectively the MIOC clinical domains (Fig. 1), utilising a phased care approach through the development and execution of personalised care pathways/plans (PCPs).^2^^,^^3^ This article intends to educate and support oral and dental health care professionals in providing such phased, person-focused, risk/needs-related MIOC for high-risk caries patients across several courses of treatment (CoT).^4^ Further guidance on opportunities for flexible commissioning in primary care dentistry in order to deliver services to meet local population needs have been published by NHS England.^5^

## Scope

Patients with high needs in England may require oral and dental care which is not always technically complex but involves a high quantity and quality of care necessary for them to personally establish control of their generalised progressive oral disease. A staged PCP prioritises disease stabilisation and prevention (which equates to environmentally sustainable care), followed by definitive restorative interventions in line with MIOC principles. This is essential to provide best clinical practice and effectiveness for patients. Recent NHS contractual changes^1^ with a staged/phased approach could support such optimal MIOC provision, rewarding clinical teams for undertaking best practise principles, potentially improving patient access and therefore helping to reduce oral and dental health inequities. Claiming fees and patient charge profiles however remain distinct issues and will require further clarification in future publications/materials. Successful MIOC implementation relies upon a team-delivered, holistic, person-focused approach, enabling care delivery to be devolved to other specialisms and team members with the appropriate skillsets.^6^ Such an approach is crucial for the management of all patients to lower/minimise their susceptibility to oral disease, especially individuals who are high-needs and high caries-risk/susceptibility patients; those who are more susceptible to developing new carious lesions and/or experience the progression of existing carious lesions, which can be established through longitudinal risk/susceptibility assessments and clinical judgement.^7^^,^^8^

## Definitions

- MIOC: best practice (environmentally sustainable), holistic, team-delivered approach to maintain life-long oral and dental health, focusing on preventive, risk/susceptibility-related, person-focused care plans and dutiful management of patient expectations. The four interlinked clinical domains of MIOC are shown in Figure 1^,^^9^^,^^10^
- PCP: offering patients/caregivers some choice and control over their care planning and delivery, based on their needs, wants and managed expectations, using goal-setting behavioural modelling and shared decision-making with oral health care teams^3^
- Phased CoT: potentially completing multiple CoT within a 12-month period, based upon the downward trend in disease risk/susceptibility of the individual patient.^4^

## Principles

Implementation of PCPs to deliver MIOC builds upon the revised note for the *Avoidance of doubt* (N4AD) guidance.^4^

## Policy

PCPs delivering MIOC facilitate provision of high-quality oral and dental care to patients with high oral and dental needs, often those who have not received routine oral and dental care in the previous two years, are living with additional general health problems or social needs, or are from more vulnerable socioeconomic backgrounds.

The communication of a unified MIOC message should be delivered by the full skill mix of the oral and dental workforce^6^ working within their defined scope of practice, including extended duties dental nurses, such as those with oral health education certification, with additional training in oral health education, plaque scoring and fluoride varnish application, dental hygienists, dental therapists, clinical dental technicians and practice administration staff, often co-ordinated and led by the principal dentist. Keeping in mind the variations and complexities of general dental practice business models, increased team member involvement enables a more efficient use of clinical time, allowing more patients to be seen daily, potentially improving access, population outcomes and improving financial and clinical rewards for team members.

The MIOC focus on risk/susceptibility-related prevention and behaviour change (both in patients and professionals) encourages the promotion of healthy oral hygiene and dietary habits, creating stable oral environments suitable for tailored long-term restorative rehabilitation and recalls.

This approach of non-/micro-/minimally invasive therapies titrated against patient response aligns fully with the general medical healthcare principles of phased personalised care pathways.^11^^,^^12^ It is also important to respect the choices and needs/expectations of patients, including those who prefer receiving immediate urgent care without maintaining an ongoing care relationship with the oral and dental health care practice.

## Phasing MIOC PCPs

**Fig. 2:**
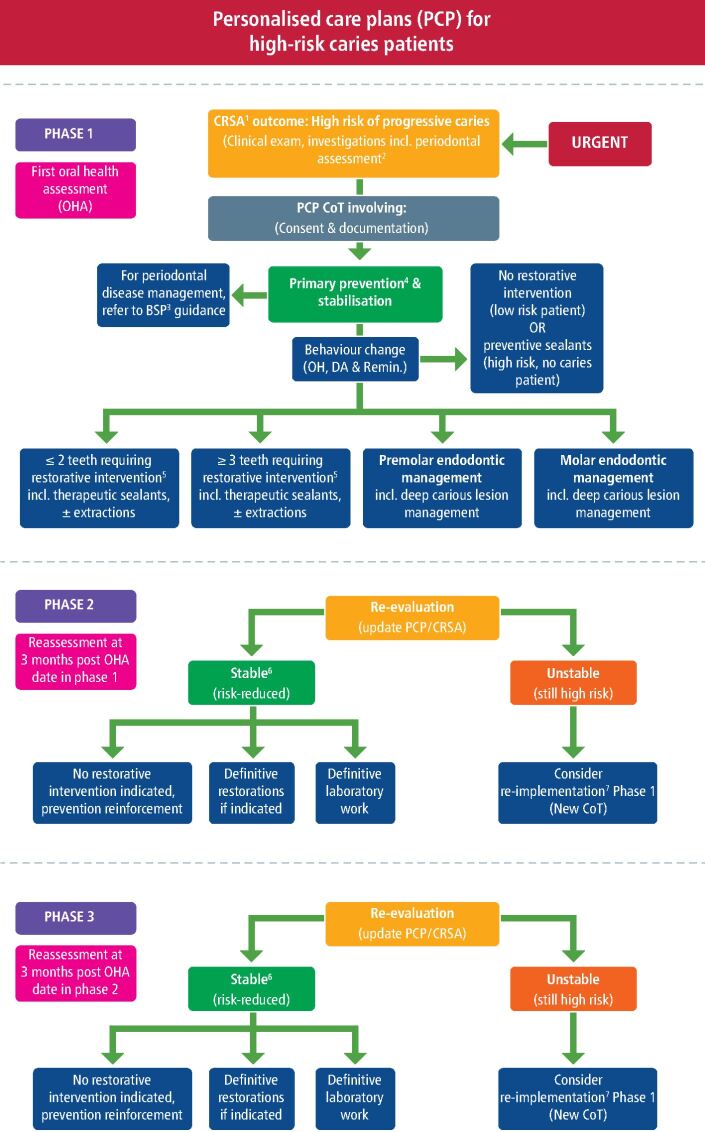
The PCP flowchart for managing high-risk/needs caries patients.^14^ This flowchart outlines the potential mechanisms of delivering MIOC through phased personalised care plans. It lists numerous types of clinical interventions available as part of a phased approach when managing high-risk/needs caries patients. Reference to the BSP S3 guidance has also been made for the management of patients with periodontal disease. (Superscripts: 1 = caries risk susceptibility assessment; 2 = soft tissue screening is expected. A tooth wear assessment can also be performed if indicated; 3 = BSP; 4 = prevention may include oral hygiene and dietary advice, remineralisation, preventive or therapeutic fissure sealants, as outlined in Delivering Better Oral Health guidelines;^13^ 5 = minimally invasive dentistry;^2^ 6 = notes for avoidance of doubt reference to stabilisation of active disease;^4^ 7 = potential circumstances exist where it would be appropriate to proceed to definitive treatment, such as indirect restorations and removable prosthesis. OH - oral hygiene; DA - dietary analysis; OHA - oral health assessment)

A comprehensive oral health assessment in the first phased CoT identifies the patient's susceptibility to various oral conditions, including dental caries, periodontal disease, oral cancer and tooth wear. For high-needs caries-susceptible patients, longitudinal caries risk/susceptibility assessments (CRSA) are essential for successful long-term holistic care. Similar principles are applied to patients presenting with periodontal disease and established guidance from the British Society Periodontology (BSP) should be considered in their management,^13^ as linked within Figure 2. Patients needing PCPl involving multiple courses of treatment should be informed at the outset that further NHS dental charges may be incurred. The precise detail and cost of subsequent phases would be unpredictable until a comprehensive clinical re-assessment consultation is completed, three months after the start date of the first phased CoT. Justifications for phasing treatment, clinical and patient factors should all be recorded contemporaneously in the patient notes, in addition to a suitably completed FP17DC, once each CoT has been planned, consented and documented.

CoT 1 starts with a full clinical oral health assessment (including examination, initial CRSA, special investigation reporting, diagnosis and prognosis) and the creation of an initial prevention-based PCP. This includes urgent pain relief (for example, temporisation of cavities, extractions of teeth with hopeless prognosis), lesion stabilisation (for example, therapeutic sealant restorations, provisional restorations using glass-ionomer cements, including occlusal load-bearing areas) and further non-operative preventive disease control measures. Some of these clinical duties can be devolved to other team members.^14^

CoT 2 re-evaluation and active surveillance protocols must align with National Institute of Care and Excellence (NICE)^7^ and *Delivering better oral health* v4^8^ guidelines. Further personalised care planning is dependent upon the patient's oral condition at re-assessment and patient consent. A level of control of oral disease must be demonstrated before progressing onto higher treatment bands, unless, for example, the need for an immediate prosthesis is indicated clinically, for example, a denture. The aim is to de-escalate the patient's risk/susceptibility status from high to risk-reduced levels through the overall stabilisation of their oral environment. This may be evaluated using longitudinal CRSA and team-delivered active surveillance. A decrease in caries incidence will reduce the need for further complex restorative treatment long-term, achieving successful long-term maintenance through the embedding of preventive habits and behaviours, all encouraging sustainable oral health care in the future.^2^^,^^9^^,^^14^ Phased CoT 2 can involve the provision of definitive functional restorations (for example, resin composites and tooth-restoration complex maintenance using the ‘5Rs' principles),^14^ providing the patient demonstrates risk-reduced stable oral health, ascertained at the re-assessment consultation appointment.

More complex interventions, surgery or indirect restorations may be more suitable in phased CoT 3, subject to the patients progress along the risk-reduced, prevention-based pathway, assessed longitudinally on recall.

## Conclusion

A personalised care plan flowchart for the management of high caries-risk patients undergoing phased care is illustrated in Figure 2. Appropriate implementation of NICE recall guidance is crucial for managing patient charge liability, while incentivising attendance and generating greater NHS capacity within the general dental services.^15^ (MeSH key terms in Box 1)

Box 1 MeSH on demand key terms

- Minimum intervention oral care
- Prevention
- Dental caries
- Disease management
- Oral healthcare
- Primary care
- Caries risk
- Caries susceptibility
- Dentistry
- Minimally invasive
- NHS
- Remuneration

## References

[1] NHS England. Outcome of 2022/23 Dental Contract Negotiations.2022. Available at https://www.england.nhs.uk/wp-content/uploads/2022/07/B1802_First-stage-of-dental-reform-letter_190722.pdf (accessed September 2023).

[2] Banerjee A. ‘MI'opia or 20/20 vision? *Br Dent J* 2013; **214:** 101-105. 10.1038/sj.bdj.2013.105 23392021

[3] NHS England. What is personalised care? Available at https://www.england.nhs.uk/personalisedcare/what-is-personalised-care/ (accessed September 2023).

[4] NHS England. Avoidance of doubt: Provision of phased treatments. 2021. Available at https://www.england.nhs.uk/wp-content/uploads/2018/02/B0615-Update-to-avoidance-of-doubt-provision-of-phased-treatments-300621-.pdf (accessed August 2023).

[5] NHS England. Opportunities for flexible commissioning in primary care dentistry: A framework for commissioners. 2023. Available at https://www.england.nhs.uk/long-read/opportunities-for-flexible-commissioning-in-primary-care-dentistry-a-framework-for-commissioners/ (accessed October 2023).

[6] NHS England. Building dental teams: Supporting the use of skill mix in NHS general dental practice - short guidance. 2023. Available at https://www.england.nhs.uk/long-read/building-dental-teams-supporting-the-use-of-skill-mix-in-nhs-general-dental-practice-short-guidance/ (accessed November 2023).

[7] National Institute for Care and Excellence. Dental checks: intervals between oral health reviews. 2004. Available at https://www.nice.org.uk/guidance/cg19/resources/dental-checks-intervals-between-oral-health-reviews-pdf-975274023877 (accessed September 2023). 39480976

[8] UK Government. Delivering better oral health: an evidence-based toolkit for prevention. 2014. Available at https://www.gov.uk/government/publications/delivering-better-oral-health-an-evidence-based-toolkit-for-prevention (accessed August 2023).

[9] Banerjee A. Minimum Intervention oral healthcare delivery - Is there consensus? *Br Dent J* 2020; **229:** 393-395. 10.1038/s41415-020-2235-x PMC7546164 33037331

[10] Heidari E, Newton J T, Banerjee A. Minimum intervention oral healthcare for people with dental phobia: a patient management pathway. *Br Dent J* 2020; **229:** 417-424. 10.1038/s41415-020-2178-2 PMC7546148 33037361

[11] National Institute for Health and Care Excellence. Type 2 diabetes in adults: management. 2015. Available at https://www.nice.org.uk/guidance/ng28 (accessed September 2023). 26741015

[12] National Institute for Health and Care Excellence. Hypertension in adults: diagnosis and management. 2023. Available at https://www.nice.org.uk/guidance/NG136 (accessed September 2023). 31577399

[13] West N, Chapple I, Claydon N *et al.* BSP implementation of European S3 - level evidence-based treatment guidelines for stage I-III periodontitis in UK clinical practice. *J Dent* 2021; **106:** 103562. 10.1016/j.jdent.2020.103562 33573801

[14] Banerjee A. *A Clinical Guide to Advanced Minimum Intervention Restorative Dentistry*. 1st ed. London: Elsevier, 2024. In press. 10.1038/s41415-024-8183-0 39672849

[15] NHS Business Services Authority. What is continuation treatment (further treatment within two months) and when would it apply? Available at https://faq.nhsbsa.nhs.uk/knowledgebase/article/KA-01998/en-us (accessed September 2023).

